# Siamese Unet Network for Waterline Detection and Barrier Shape Change Analysis from Long-Term and Large Numbers of Satellite Imagery

**DOI:** 10.3390/s23239337

**Published:** 2023-11-22

**Authors:** Hsien-Kuo Chang, Wei-Wei Chen, Jia-Si Jhang, Jin-Cheng Liou

**Affiliations:** 1 Department of Civil Engineering, National Yangming Chiaotung University, Hsinchu 300, Taiwan; hkc@nycu.edu.tw (H.-K.C.); jcliou@nycu.edu.tw (J.-C.L.); 2 Synopsys Incorporated, Hsinchu 300, Taiwan; jayce@synopsys.com

**Keywords:** deep-learning network, area attenuation, barrier change, satellite-derived shoreline

## Abstract

Barrier islands are vital dynamic landforms that not only host ecological resources but often protect coastal ecosystems from storm damage. The Waisanding Barrier (WSDB) in Taiwan has suffered from continuous beach erosion in recent decades. In this study, we developed a SiamUnet network compared to three basic DeepUnet networks with different image sizes to effectively detect barrier waterlines from 207 high-resolution satellite images. The evolution of the barrier waterline shape is obtained to present two special morphologic changes at the southern end and the evolution of the entire waterline. The time periods of separation of the southern end from the main WSDB are determined and discussed. We also show that the southern L-shaped end has occurred recently from the end of 2017 until 2021. The length of the L-shaped end gradually decreases during the summer, but gradually increases during the winter. The L-shaped end obviously has a seasonal and jagged change. The attenuation rate of the land area is analyzed as −0.344 km^2^/year. We also explore two factors that affect the analysis results, which are the number of valid images selected and the deviation threshold from the mean sea level.

## 1. Introduction

Barrier change is a complex evolutionary process of coastal topography, which attracts some scholars’ interest in exploring its mechanism. The Waisanding Barrier, which is oriented sub-parallel to the west coast of Taiwan, has suffered continuous beach erosion in recent decades. The traditional method to understand the change in beach bathymetry is to calculate the difference in bathymetrical elevation from pre- and post-site measurements. In the early stages, the work of such elevation maps obtained from land surveys and sea surveys is quite time-consuming, and labor intensive. If the research focuses on land geomorphology and does not involve the sedimentation mechanisms for beach erosion or accretion and seabed topography changes, satellite image analysis combined with LiDAR technology is a fast, convenient and low-cost method. LiDAR can offer an incredibly accurate and consistent digital elevation model that can make high-resolution maps with applications in surveying, geodesy, and geomorphology. In recent decades, some research has been conducted to analyze the shoreline changes of the WSDB [1,2,3]. These studies used several years of satellite imagery, LiDAR measurements, or in situ topographic survey data to analyze shoreline changes, so these studies only provided the characteristics of the short-term changes of WSDB during the data period, but it is difficult to fully understand its long-term changes.

Traditionally, the collection of traditional shoreline datasets is often expensive and constrained in time and/or space. Until recently, obtaining accurate shorelines over large geographic areas from high-resolution satellite imagery provided applicable shoreline mapping for further studies on shoreline evolution. The full collection of satellite images and the use of state-of-the-art image processing techniques are available to automatically derive a satellite-derived shoreline position from satellite imagery without disturbances such as cloud cover, foam caused by surf, and atmospheric interactions [4]. By quantifying the positional accuracy of satellite-derived shoreline positions, we can easily and visually understand the evolution of the shoreline or the change in shape of a barrier [5,6,7,8,9,10].

Accurate analysis of shoreline changes is necessary and basic work for the academic exploration of island topographic changes and engineering applications. The location of the shoreline is derived from in situ topographical surveying, which is based on common data. However, the interaction of the sea and the beach is commonly defined as the waterline or shoreline at some tidal level, which is extracted from aerial photography, video imagery, or satellite imagery. For remote sensing imagery, the detection of sea–land segmentation is to separate a shoreline image into ocean region and land region. The waterline on the beach varies in time due to semi-diurnally or diurnally periodic tide levels and is different from the zero-meter shoreline based on mean sea level. For a precise study of the evolution of beaches using shoreline changes, the original shorelines extracted from images at different periods should be transferred to zero-meter shoreline and then the change in the zero-meter shoreline should be analyzed [11]. The purpose of this study is to use multiple images to explore long-term changes in the shape of the WSDB.

Previous studies on the satellite-derived shoreline position are often limited by the large number of clear images used. An automated or semi-automated technique to delineate the zero-meter shoreline from the waterline of an image in the classifier has become an urgent need to be developed in order to cope with a large number of image processing. The waterline extraction of an image is object detection in image processing. Convolutional neural networks (CNNs) provide a translation invariance feature to maintain the spatial information of the image so that CNN can effectively recognize the features of adjacent images. CNN is examined to be one of the best learning algorithms due to its superior performance in the fields of image processing and computer vision [12,13]. CNNs have been successfully applied to different machine learning-related tasks, such as object detection, recognition, classification, regression, and segmentation [14,15,16]. Furthermore, CNNs have been applied in medical image analysis [17,18,19,20] and machine vision, classification of plant diseases, etc. [21,22,23].

UNet, first developed by Ronneberger et al. [24], is a neural network architecture designed primarily for image segmentation. There are several variants that aim to improve performance compared to the original UNet architecture. For sea–land segmentation, a DeepUNet with DownBlocks instead of convolution layers was proposed in the contracting path and uses UpBlocks in the expansive path to obtain more precise images of both areas. Siddique et al. [25] examined the major deep learning methods and their application areas. The effect of patch size and network architecture on a CNN approach for automatic segmentation was discussed [26]. We consider the high utility of DeepUNet with three types of patch sizes in sea-land segmentation for this study.

Recently, the multilayered, hierarchical structure of deep CNN has the ability to extract low-, mid-, and high-level feature. The high performance of deep CNN in recognition tasks has been shown due to its hierarchical ability to extract features to deal with complex learning problems [27,28]. The performance of CNNs has improved the accuracy of object recognition as the depth of CNNs has increased [29,30,31]. The availability of big data and advancements in hardware are also the main reasons for the fast-increasing applications of deep CNNs in wide fields [32,33,34,35]. However, some challenges, including the necessity of powerful hardware resources, poor estimation of the pose, orientation, and location of an object, high influence of hyper-parameter section on the performance, etc., are still required to be overcome in the future. The intrinsic taxonomy and prominent architectures of CNNs were reviewed by [9,12,36].

Based on the advantage of CNN with high image recognition ability and the aim of this study, we develop an applicable Siamese neural network compared to the DeepUNet network with different sampling sizes for waterline detection from an image. Siddique et al. [25] examined the main deep learning methods and their application areas for all articles on image modalities and reviewed the many variants of DeepUNet and its diverse applications on a multitude of image segmentations. We consider the high utility of DeepUNet in image segmentation as the primary network architecture. In addition, to ensure that DeepUNet can learn that patch image samples are part of the WSDB, we combine a basic UNet network with a Siamese network, also called a twin network, to provide additional information about the overall shape of the WSDB. Bhatt et al. [36] compared various architectural evolutions in CNN by its architectural change, strengths, and weaknesses.

A Siamese neural network, also called a twin neural network, was first developed by Bromley et al. [37] to solve signature verification as an image matching problem. A Siamese network employs a unique structure to naturally rank the similarity between inputs. Through the uses of similarity measures, a Siamese network has been widely used for recognizing handwritten checks, automatic detection of faces in camera images, and matching queries with indexed documents, using one-shot image recognition and object tracking. The Siamese network mainly aims to overcome the problem that UNet cannot fully identify the correct image when the brightness of the sea part of the image is too bright or the brightness of the sea and land is relatively special. After first being introduced in 2016, the Siamese architecture was employed to improve the localization accuracy of deep trackers in many high-performance applications for the problem of real-time object tracking or change detection. These Siamese networks are SiamFC [38], DaSiam [39], SiamRPN++ [40], CIR [35], C-PRN [41], DASNet [42], LSNET [43], and Siamese_AUNet [44]. For a review of multi-class change detection for satellite remote sensing imagery please refer to Zhu et al. [45]. Remote sensing image change detection is used to identify interesting differences in land characteristics by comparing multiple images over time. For the problem of WSDB, we developed a new SiamUnet network to classify and determine the spatial distribution of land and water of an individual image. These changes in the land–water boundaries between two images may not be real but caused by wave foam and water puddles on land. In order to avoid the above unreal changes and ensure the accuracy of subsequent analysis, we manually adjust the pixels in this part of the image to determine whether it is land or sea. Only then can we correctly evaluate the network recognition accuracy of the land area in each image and confirm the subsequent analysis results of land area changes.

In order to investigate the long-term change in shape of the WSDB barrier, we selected 207 available SPOT 5–7 high-resolution satellite images from 2004 to 2021. We introduce the WSDB and the data used in Section 2.1. In Section 2.2, we describe the necessary image processing for 207 collected clear images and three DeepUNets with different sampling sizes and a combined SiamUnet. The performance evaluation of the proposed network is presented and the annual change in the land area of the WSDB is discussed in Section 3. In Section 4, the factors affecting area attenuation are discussed, along with the limitations and future developments of SiamUnet in this study. Finally, we summarize our findings in Section 5.

## 2. Materials and Methods

### 2.1. Materials

**Figure 1:**
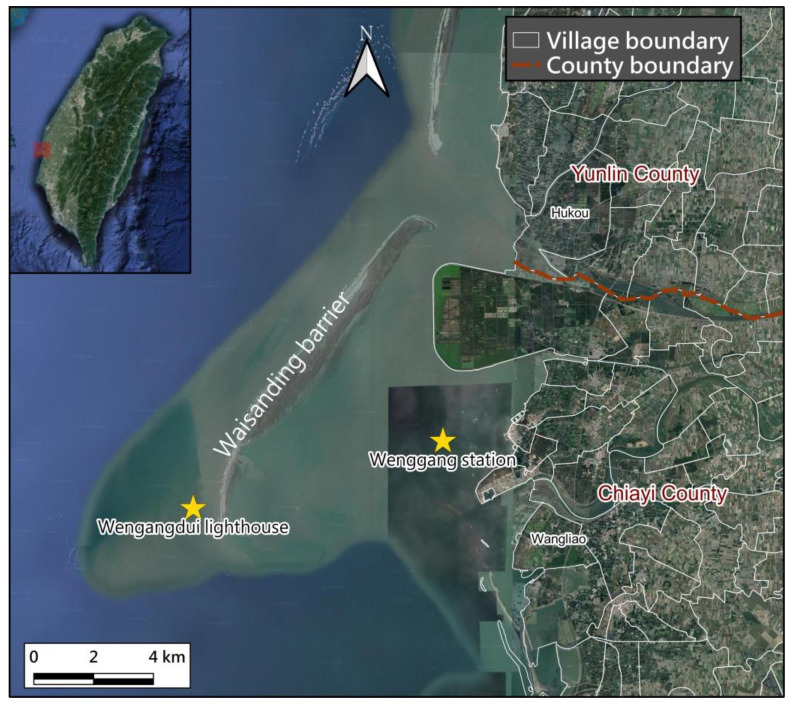
Location of the WSDB in a cut and reproduced Google Earth map from 2020.

To provide the latest and highest resolution map of the study site, we use QGIS 3.16 software and mapping services that present data by stitching. Figure 1 shows the location of the WSDB on the Google Earth map for 2020. The WSDB is a long, thin, low-lying, sandy barrier island and is the largest barrier in Taiwan. It extends from the coast of Hukou Village in Yunlin in the southwest to the sea off Wangliao Village in Chiayi. It reaches 12.6 km and its land area is approximately 12.5 square km at low tide (about −1.2 m relative to the mean sea level). The WSDB is oriented subparallel to the west coast of Taiwan. The low-speed lagoon between the WSDB and Taiwan’s land is the main water area for oyster farming in Taiwan. However, the WSDB has suffered from continuous beach erosion and has gradually disappeared in recent decades.

The tidal range here can be estimated from the Wenggang gauge station located between the WSDB and Taiwan and is about 150 cm, which is obtained from the statistical analysis of marine data on the website of the Central Meteorological Bureau in Taiwan. During the period from the southwest monsoon to the northeast monsoon, the observed offshore wave heights of WSDB are relatively large. The main wave heights are between 50–150 cm on average, and the main period is between 4–7 s in this water.

We used a hierarchical cluster method to classify the two seasons of monthly average wind speed components as summer from May to August and winter from September to April of the following year. If the wind direction is used as classification data, the wind direction distribution from May to September is relatively close and can be divided into summer, with October to April of the following year being winter.

A total of 207 clear images with few clouds above WSDB were collected from SPOT-5 and SPOT-6 and 7 satellites from 2004 to 2020. The early images of the SPOT series have low resolution. The resolution of the panchromatic and multispectral images taken by the SPOT-5 satellite launched in 2003 can reach 5 m and 10 m, respectively. The spatial resolution and the number of different satellite images selected are shown in Table 1.

### 2.2. Methodology

#### 2.2.1. Image Pre-Processing

The image processing techniques involve six steps, which are (1) pan-sharpen; (2) calculation of the normalized difference water index (*NDWI*) to convert image information; (3) image enhancement; (4) morphology image processing for combination operations such as expansion or erosion; and (5) binarization of land cover and sea cover markers.

Panchromatic images have higher spatial resolution than multispectral images, but multispectral images have extensive reflection information in different bands. To combine the two advantages, image fusion can be used to improve the resolution of multispectral images. The related methods include HSI, Wavelet, PCA and other methods. We use the common and simple HSI method.

In recent years, the literature on remote sensing for identifying the coast and beach has pointed out that the Normalized Difference Water Index (*NDWI*) is a good indicator to detect the boundary between water and land in the coastal zone [46,47,48]. *NDWI* is chosen for the classifier index in shoreline detection from multispectral imagery. The definition of *NDWI* is

(1) $$NDWI=\frac{Green-NIR}{Green+NIR}$$

where *Green* is green light and *NIR* stands for near-infrared light.

Compared with the general waterline with a clear boundary between water and land, the spectral difference between shallow water and wet sand around the wetland environ-ment is not large, so it is quite difficult to find a suitable threshold to distinguish the two ranges even through visual examination. The problem can be solved using image enhancement, which refers to the process of highlighting certain information of an image, as well as weakening or removing any unnecessary information according to specific needs. Image enhancement extends the original input luminance values in all grayscale values to take advantage of the total dynamic range. The algorithm can refer to Jain and Singh [49].

Morphological image processing is a collection of non-linear operations related to the shape or morphology of features in an image [50]. Morphological operations rely only on the relative ordering of pixel values, not on their numerical values, and therefore are especially suited to the processing of binary images. Morphological operations are commonly applied to binary images that may contain numerous imperfections, such as noise and textures, which can be distorted when binary regions are produced by a simple threshold. Morphological techniques are used to probe an image with a small shape or template called a structuring element. The structuring element is positioned at all possible locations in the image and compared with the corresponding neighborhood of pixels. Dilation and erosion are two fundamental operations in morphological techniques. The dilation operation in the assigned structuring element is used to probe and expand the shapes contained in the input image. In contrast to dilation, the assigned structuring element is used to probe and reduce the shapes contained in the input image in the erosion operation. We applied morphological image processing to fill all small water-cover areas that may be corrected to the land-cover part of the WSDB.

By means of image binarization, the image can be efficiently and rapidly segmented, allowing for the demarcation of the boundaries between sea and land areas. This method enables accurate division of these areas, facilitating subsequent analysis and modeling processes. Otsu [51] provided a popular method for empirically determining the dual threshold value. This method generates the high threshold on a non-maximum suppressed gradient magnitude image. The low threshold is typically set to 1/2 of the high threshold in this case. Otsu’s automatic thresholding method has the advantage of adaptively using value/count pairs rather than full histograms, however, some cases still require manual intervention for correction.

After the above image processing, the land and water cover of the WSDB in the image can be distinguished as a binarized image, also known as a black-and-white image, which can be marked as 0 and 1, respectively. The boundary of the WSDB land area is the waterline at the time of the image. The generated binary images are utilized as ground truth data for subsequent deep learning. Meanwhile, the corresponding original spectral bands, inclusive of red, green, and near-infrared bands, are employed as the input data for ensuing deep learning tasks.

A huge amount of data is commonly suggested to build an accurate deep learning model. Some popular publicly available image datasets are available for medical image segmentation tasks shown in the Appendix of Siddique et al. [25]. However, no popular benchmark dataset is available for the extraction of waterlines in the coastal zone. The number of image patches with 256 × 256 sliced from a whole frame image is about 526. The total number of image patches is 105,726. However, the number of land cover image patches represent about 12.41% of the total. Most of the patches are in the water cover image. Such image samples with disparate proportions may cause misjudgments in model training. Resampling is a method to solve the problem by adjusting the imbalanced dataset to be close.

The undersampling method is used to reduce the number of similar samples. A total of 80% of the water cover image patches are randomly sampled in this study. For a few land cover samples, we use the oversampling method to increase the number of enhanced data. Data augmentation includes image amplification, random size capture, horizontal flip, vertical flip, random rotation, and elastic deformation. The principle effect of this increase in data can be found in many previous research results [30,52]. The probability of each augmentation is set to 50%. The number of augmented images is four times the number of the original images. The ratio of the land cover dataset to the water cover dataset is approximately 0.78.

#### 2.2.2. DeepUNET

**Figure 2:**
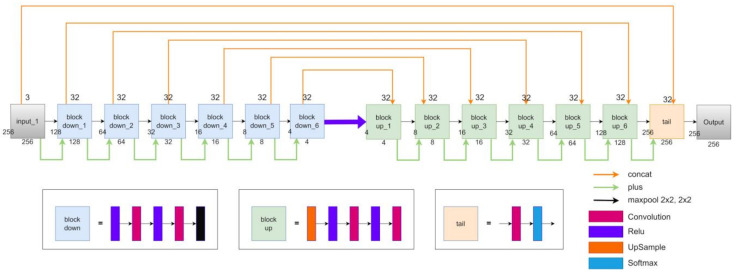
DeepUNet architecture.

Semantic segmentation is a crucial research topic in the field of optical remote sensing image processing. Segmentation of the sea and land areas is particularly challenging due to the complex and dynamic maritime environment. Although neural networks have shown excellent performance in semantic segmentation in recent years, few studies have focused on applying convolutional neural networks (CNNs) to sea–land segmentation, and further improvements in performance are possible. Li et al. [53] proposed a novel deep convolution neural network named DeepUNet. Like the U-Net, its structure has a contracting path and an expansive path to obtain high-resolution optical output. On the other hand, the DeepUNet uses DownBlocks instead of convolution layers in the contracting path and UpBlocks in the expansive path. The two novel blocks bring two new connections that are the U-connection and the Plus connection. They are promoted to obtain more precise segmentation results. The DeepUNet architecture is shown in Figure 2.

#### 2.2.3. SiamUnet

**Figure 3:**
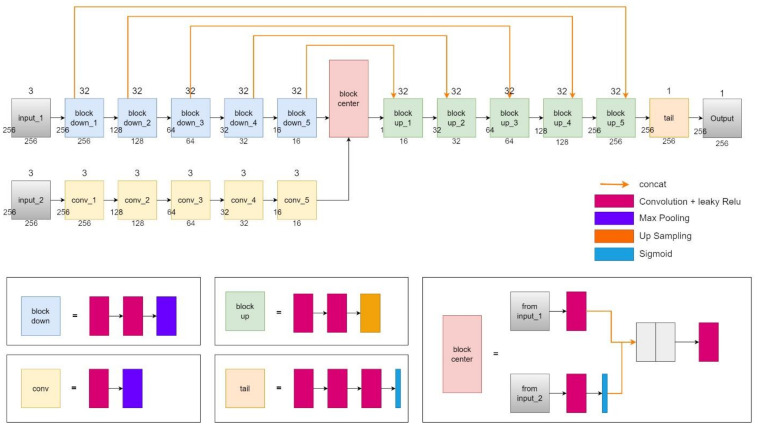
The proposed SiamUnet architecture. The input_1 is the UNet architecture with sliced image inputs and the input_2 is the input with the whole WSDB image.

Figure 3 illustrates the SiamUnet architecture, we use U-net on small-scale high resolution images to perform pixel-to-pixel segmentation, and use a light fully CNN on large-scale low-resolution images to obtain large scale information. The basic structure of the UNet architecture consists of two paths that are a contracting path and an expansive path. The contracting path follows a typical CNN architecture. Each block in the contracting path consists of repeated application of two successive 3 × 3 convolutions, each followed by a rectified linear unit (ReLU) and a 2 × 2 max-pooling layer for downsampling. ReLU is a fast learning activation function and is one of the most successful and widely used activation functions [9,13]. At each downsampling step, we keep the number of feature channels.

The second part of UNet is the expansive path, in which each block consists of two successive 3 × 3 convolution layers followed by an upsampling layer used for deconvolution. For each up-block, the feature map of the corresponding layer in the contracting path is concatenated. At the final stage, an additional tail unit is used to reduce the feature map to the required number of channels and produce the segmented image. Therefore, this expansion allows the network to learn localized classification information and increases the resolution of the output, which can then be passed to a final convolutional layer to create a fully segmented image.

The resulting network is almost symmetrical, resembling a U-shaped shape. More importantly, UNet propagates contextual information along the network, which allows it to segment objects in an area using context from a larger overlapping area.

Through a comparison of input sample sizes, it has been found that models that utilize larger input areas yield better inference results. Therefore, to maintain the original high-resolution, it is advisable to use large-scale samples as input whenever possible. However, this approach demands substantial computational resources. To address this challenge, a Siamese-Unet method is proposed. In this approach, a U-net architecture is used for pixel-to-pixel segmentation on small-scale, high-resolution images, while a fully convolutional neural network (CNN) is utilized for capturing large-scale information on large-scale, low-resolution images.

#### 2.2.4. Performance Evaluation

When the proposed SiamUnet reaches the minimal loss depending on the difference between the predictions and the training samples, and the loss is acceptable in the verification samples, the final architecture is determined. Whether or not the well-trained model has the problem of overlearning, it must be examined for application and performance in the training stage.

The confusion matrix, also known as an error matrix, is a very popular measure used to evaluate the performance of classifiers [54]. A confusion matrix is a table with two rows and columns in which the number represents the counts from the predicted and actual values [55]. These four outputs are named TP, TN, FP, and FN. TP stands for true positive, which indicates the number of positive examples classified accurately. Similarly, TN stands for true negative, which shows the number of negative examples classified accurately. FP shows a false positive value, which is the number of actual negative examples classified as positive and FN means a false negative value, which is the number of actual positive examples classified as negative.

Based on the confusion matrix stated above, precision, recall, and F1 score are calculated and commonly used for the performance of an algorithm [55,56]. Accuracy gives the proportion of the total number of predictions as defined by the ratio of TP + TN to TP + FP + TN + FN. Accuracy is a great measure, but only when you have symmetric datasets; also, false negatives and false positives have similar costs. The precision is defined by P = TP/(TP + FP), indicating the fraction of positive values from the total predicted instances. The definition of recall, also called sensitivity, is the proportion of actual positive cases that are correctly identified, showing the fraction of positive values out of the total actual instances. The definition is R = TP/(TP + FN). Precision is generally used when the positive values in the predicted values are more important than the actual values, and recall is used in the opposite situation.

The IoU (Intersection over Union) score gives the similarity between the predicted region and the ground truth region for an object present in the set of images and is commonly used as a standard measure for the object category segmentation problem. Rahman and Wang [57] proposed an alternative IoU score, defined as Equation (2), using the probability values.

(2) $$IoU=\frac{I(X,Y)}{U(X,Y)}$$

where *X* is the output of the network, *Y* is the ground truth data, *I*(*X*, *Y*) is the area intersection of the predicted output and the ground truth assignment set, and *U*(*X*,*Y*) is the area union of both sets. The probability that a Region-of-Interest (ROI) object is correctly classified on the basis of its entirety should be large. On the other hand, it should be small if it is classified only based on its part. Thus, if an ROI has a large IoU overlap with its corresponding Ground-Truth box, then the ROI should be punished more when it is misclassified. From the definition of IoU, Equation (2), it is very easy to understand that the higher the IoU, the better the network performance.

For the problem of object segmentation, the pixels of an image (foreground) for the specified object are much less than the other pixels (background). In such a problem setting, two classes are often very imbalanced, since most of the pixels in an image usually belong to the background. Using indicators such as P, R, and F1 scores to evaluate overall network performance may fall into the disadvantage of learning most of the background without accurately identifying a few pixels of the object. The IoU measure can take into account the issue of class imbalance and is usually present in a situation such as this [57]. Yan et al. [58] developed an effective cascade R-CNN to extract the ROI feature in remote sensing imagery by applying the IoU-based weighted loss during training. Evaluating the network performance of WSDB segmentation is also an imbalanced problem setting. The IoU measure is more suitable than the P, R, and F1 scores. Therefore, this study mainly uses IoU to evaluate the performance of the network.

**Figure 4:**
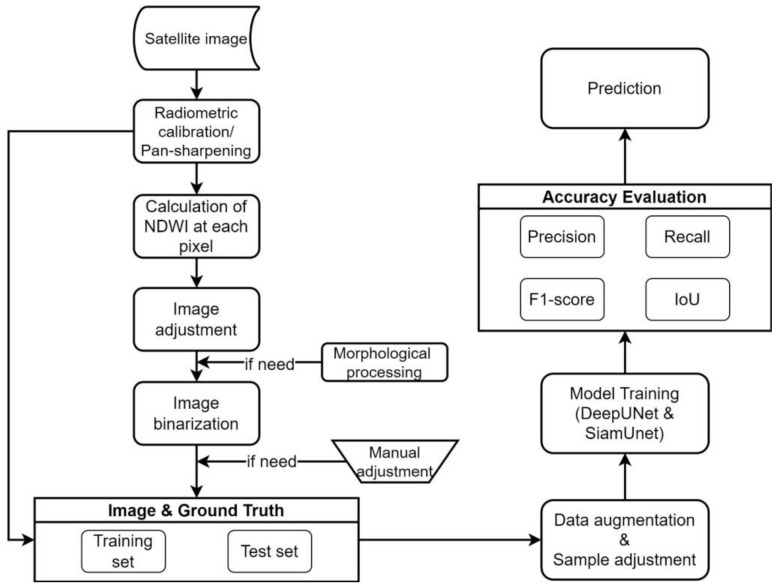
The flow chart of this study.

Figure 4 shows the flow chart of this study. The first stage was the data preprocessing mentioned in Section 3. The labeled data undergo segmentation into image blocks which are then divided into two datasets: the training set and the test set. The allocation ratio for these sets is established at 90% and 10%, respectively. Using this ratio, we ensure that the model is trained on a sufficient amount of data while allowing for robust testing of its performance. The segmentation process is automated, reducing the need for manual intervention and improving the efficiency and accuracy of the training and testing processes. Next, three UNets with different patches and a SiamUnet were constructed and trained. At the stage of precision evaluation, the trained models were quantitatively evaluated by the IoU. 

## 3. Results

### 3.1. Model Accuracy Evaluation

We analyze whether the overall shape information of the WSDB provided by the Siamese network can increase the performance of the basic DeepUNet network for image segmentation in this study. The ground length of each full-frame image is approximately 17 km. The full-frame image size is 6800 × 6800. The calculations required to build the networks are to be performed on personal computers. Due to memory limitations, it is not possible to directly use a full image of the entire area so the image needs to be sliced into small patches. The impact of patches of different sizes on recognition accuracy is shown in this section.

Three kinds of sliced patches from each full-frame image were used as training samples to train the DeepUNet network. The image size of these patches is 256 × 256, 512 × 512, and 1024 × 1024. The pixel resolution of the selected image is 2.5 m, so the ground size of a 512 × 512 image is about 1.28 km × 1.28 km. The length of the WSDB is about 12 km and the width is about 1 km. So, a patch with 512 × 512 pixels is about one tenth the size of a WSDB. The networks trained with different sample sizes are called DeepUNet-256, DeepUNet-512, and DeepUNet-1024, respectively.

**Figure 5:**
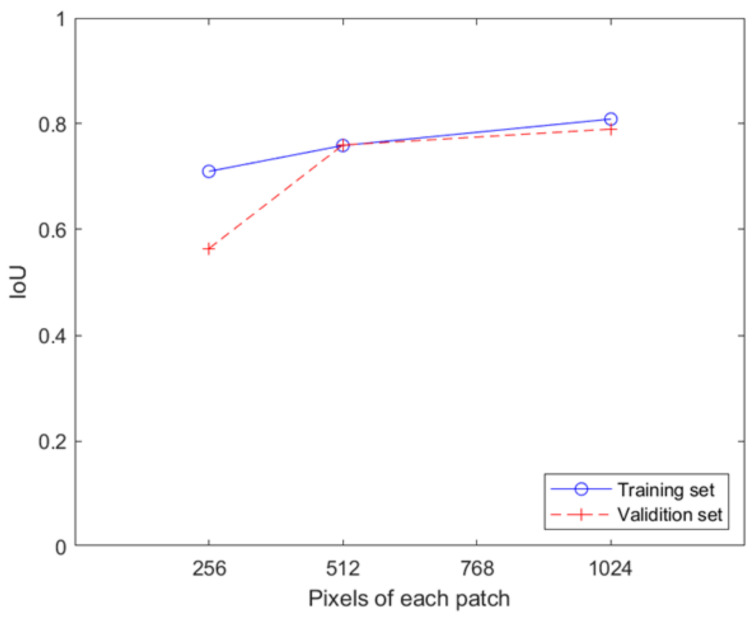
Mean IoU of all training and validation images for three DeepUNet networks.

We calculate the IoU score of land pixels identified by a well-trained DeepUNet using patches of different sizes. The mean IoU of all training and validation images for three DeepUNet networks is shown in Figure 5. It can be seen in Figure 5 that the mean IoU increases approximately linearly with the pixels of each patch from 0.71 to 0.80 in the training test and is at a low value of 0.56 for DeepUNet-256 and up to 0.79 for DeepUNet-1024 in the validation test. The result shows that both DeepUNet-512 and DeepUNet-1024 can learn to recognize the sea and land parts of the image. The IoU of the validation samples for DeepUNet-256 is significantly lower than that of the training samples for the recognition errors for DeepUNet-256 in the test.

Figure 5 also shows that the mean IoUs of DeepUNet-512 and DeepUNet-1024 for the training set are slightly higher than those of the validation set, except for DeepUNet-256. This common result shows that DeepUNet-512 and DeepUNet-1024 do not have an overlearning problem and have been well trained to reliably identify the land and sea parts of the image.

To carefully compare the performance of these networks, the IoU statistics for 207 images are listed in Table 2. Q1, Q2, and Q3 denote the first, second, and third quartile, respectively, and std indicates the standard deviation of all IoU values. Q2 is the median of the ordered IoU from smallest to largest. If Q2 is used to divide the ordered dataset into two halves, Q1 is the median of the lower half of the data and Q3 is the median of the upper half of the data. A network with a higher quartile and maximum IoU values in Table 2 indicates that the network has a higher precision in the detection of sea–land segmentation. The low std of all IoU values shows that such a network has high precision for all images.

All statistics of DeepUNet-256 in Table 2 compared to those of the other three networks show the worst performance among the four networks. The result indicates that the samples used by DeepUNet-256 are too small to learn the overall shape of the WSDB, so the segmentation detection of some images is not good, and the SiamUnet is proven to improve the accuracy of segmentation detection for most images by DeepUNet-256 through the general WSDB shape information provided in the Siamese network.

Table 2 also shows that Q2 and Q3 obtained with the SiamUnet are higher than those with the DeepUNet-512 and DeepUNet-1024, but Q1 with the SiamUnet is lower than with the DeepUNet-512 and DeepUNet-1024. Therefore, the std of SiamUnet is higher than that of DeepUNet-512 and DeepUNet-1024. When DeepUNet-256 segmentation detection is too poor for some images, the improvement by SiamUnet is limited.

From the statistics in Table 2 it can be seen that SiamUnet provides slightly better segmentation detection of the WSDB than DeepUNet-512 and DeepUNet-1024.

### 3.2. Evolution Diagram of All Detected Waterlines

**Figure 6:**
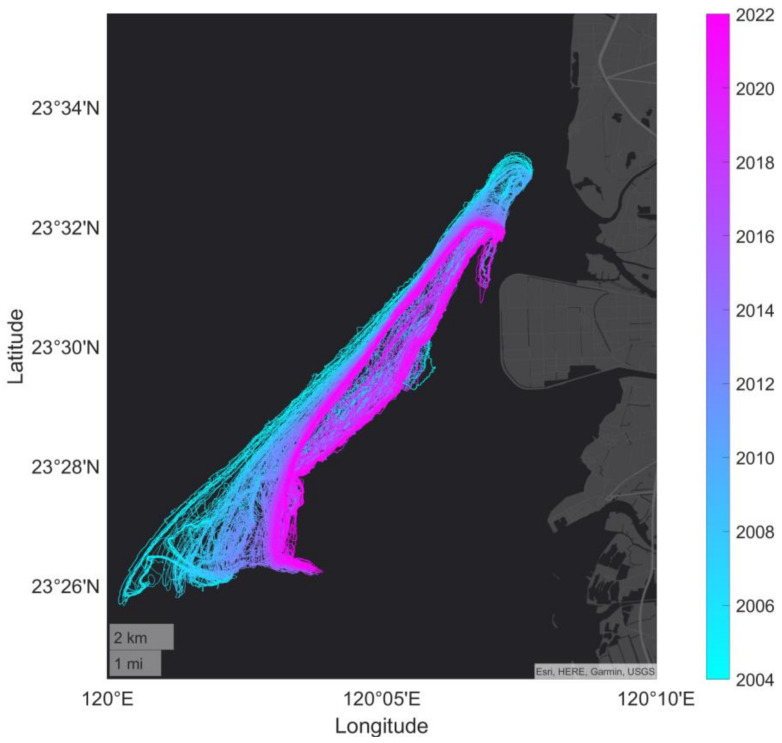
Shape diagram of 207 detected waterlines of the WSDB barrier.

We completed the detection of the WSDB waterline in 207 images from 2004 to 2021 through the proposed AI waterline identification model and some manual interventions, as shown in Figure 6. Color diagrams from cyan to magenta represent all waterline positions from 2004 to 2021. Although the tide level at the time when the image was taken may be different, on the whole it is easily seen from such a large number of waterlines that both ends of the WSDB have gradually shrunk inward over the past 18 years. It shows that the length of the WSDB is shortened. Another obvious shape shows that WSDB is gradually declining and that the beach orientation will be parallel to the land of Taiwan. Since these detected waterlines are not at the same elevation datum, it is difficult to correctly estimate the movement speed of the zero-meter shoreline, which is worthy of further study.

**Figure 7:**
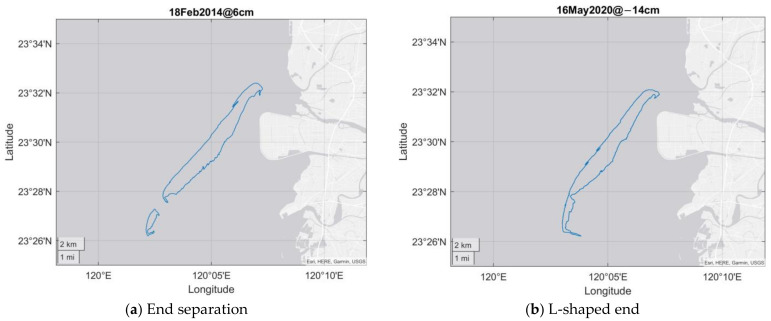
Example of the separation and L-shape of the southern end of the WSDB.

In addition, there are two minor changes that occur from time to time at the southern end. The first phenomenon is that the southern end separates from the whole barrier. It happens when the width near the southern end is sometimes quite narrow and its bottom is washed over by the sea to form a channel. The second phenomenon is that the southern end will turn sharply during a certain period in recent years to form an L-shaped end. Figure 7a,b present the cases of two results. Figure 7a is for the end separation of the image on 18 February 2014. The tidal level is 6 cm. Figure 7b is for the L-shaped on 16 May 2020, for which the tidal level is −14 cm.

The phenomenon of separation and combination of the southern part of WSDB occurs one after another. The time, conditions, and mechanism of this separation and combination are subjects of academic value and will be investigated in the next subsection.

### 3.3. Separation of the Southern End

Separation of the southern WSDB has been found in earlier in situ elevation surveys from 1984 to 2020. The report states that since 2013, a ditch has formed about 5 km near the southern end of the WSDB. When the ditch gradually widened and deepened, the southern WSDB appeared to be separated from the entire WSDB at high tide even near the mean sea level. In 2018, the ditch was filled due to sand supply, but a new ditch was generated about 2 km north of the original ditch.

**Figure 8:**
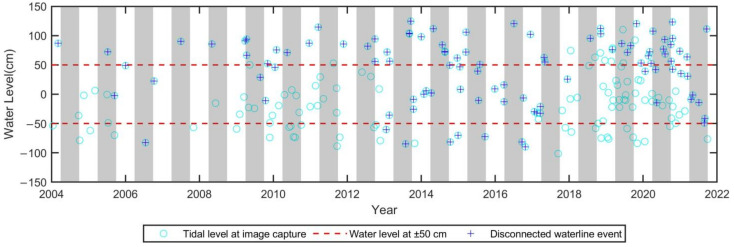
Water level at the time when the satellite image was taken.

Because the time interval between two consecutive measurements is at least half a year or even over several years, it is difficult to exactly clarify the occurrence time of the ditch. This problem can be solved using multiple images that are much denser than in-site bathymetrical observations. The open circles in Figure 8 show the corresponding tidal level at the time that each satellite image was taken. In Figure 8 the grayscale bars represent images taken in summer, and two dashed lines indicate the water level at ±50 cm. When the detected waterline is disconnected, the case with a small and closed contour, as in Figure 7a, is represented by the cross. This situation with a cross in Figure 8 shows the southern WSDB that forms an isolated part of the main WSDB.

Because the elevation of the highest land area in the southern WSDB slightly exceeds MWL, the mean sea level the case of the crosses in Figure 8 accounts for most of those above 50 cm, but there are some exceptions between 2018 and 2020. The result indicates that there is a ditch, whose bottom is less than 50 cm, near the southern WSDB and the top land to the southern WSDB is still higher than 50 cm except for the period from 2018 to 2020.

For the situation of a water level less than 50 cm, some crosses occurred in 2005, 2006, 2009, 2013–2018, and 2021. The result for the period of 2013 to 2018 has been mentioned in previous reports using in situ elevation surveys. The crosses below the dashed line of the lower limit in Figure 8 show that during this period, the bottom of the ditch was scoured deeper after the end of summer 2013, reaching nearly −1 m, until the end of summer 2017 and then deposited up to about +0.5 m in the winter of 2017. Furthermore, we also found that southern separation also occurred at the end of summer in other short-term periods. Especially in summer 2006, the bottom of the ditch dropped to nearly −1 m in middle of the summer of 2006, and then quickly rose at the end of that summer.

### 3.4. L-Shaped End

The forebeach in the swash zone is a smooth surface without complex topography, and the shoreline can be accurately interpolated to the mean sea level from some level contours, which are the detected waterlines from selected images. To study the length of the L-shaped end of the southern barrier at the mean sea level, we first selected satellite images from 2016 to 2020, when the tide level is within ±50 cm. Very few images were taken when the tide was at zero level. Therefore, we used the waterlines of some short-term images to construct the topographic contour map. We used three criteria to select these images to make reliable contours. The first criterion is the shooting time of the selected images within a period of 45 days. This consideration is mainly due to the sufficient number of available images to draw some level contours and possible slight changes in the beach profile so as not to alter much of these contours during the 45 days. To ensure the accuracy of the shoreline obtained by interpolating these contours rather than by extrapolation, we set more conditions for these contours. The contours of the first quartile level must be less than −30 cm and those of the third quartile higher than 30 cm. The third criterion is that the mean of the level contours is within ±30 cm.

**Figure 9:**
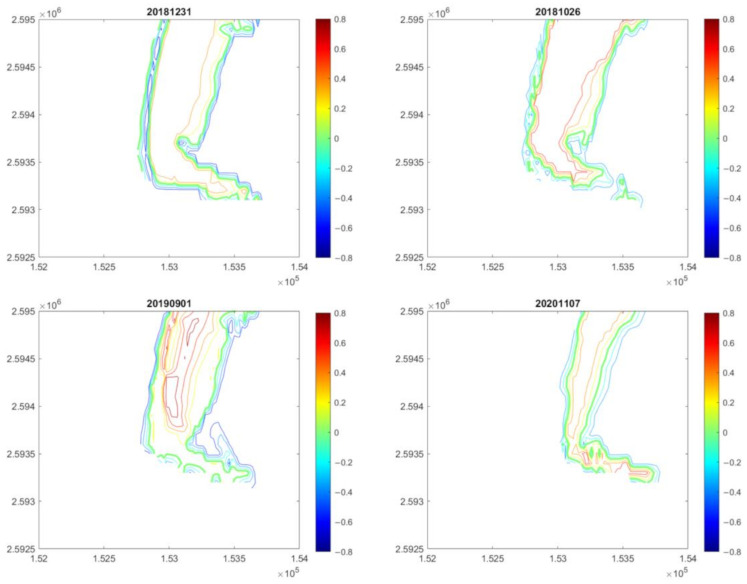
Four examples of constructed topographic contour maps. The time is indicated on the top of each panel.

Based on the above conditions, we finally constructed a total of 15 topographic contour maps. Four examples are shown in Figure 9. We used these contour maps to determine the contour of the zero-meter shoreline. We used the central line of the 0 m contour as the length of the turning part at the L-shaped end.

**Figure 10:**
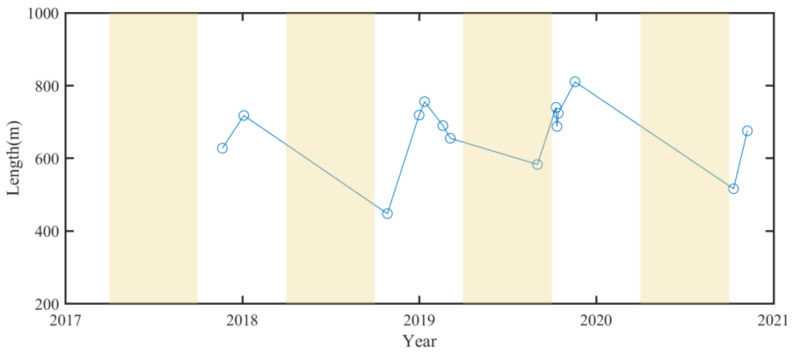
Annual variation of the length of the southern L-shaped end. The circle is the length of the southern L-shape end in the image, and the line connecting the two circles represents the linear change of both L-shape lengths with time.

Based on these constructed bathymetric maps, we can interpolate the position of the 0 m shoreline, and then determine the length of the L-shaped end. The result from 2017 to 2021 is shown by open circles in Figure 10, in which the light-yellow background is for summer and the other for winter.

Figure 10 shows a zigzag yearly variation of the length of the end of the L-shaped end. This length gradually decreases in summer, but gradually increases in winter. The variation of the length is obviously seasonal. From the above analysis, it can be determined that the L-shaped end has existed since the end of 2017 until 2021 and has seasonal changes. Because the collected images are only up to 2021, it is impossible to know whether the L-shaped end of the southern WSDB still exists.

From the analysis of a large number of satellite images, we can better determine the time when the turning end of the L-shaped end occurs and its length changes with time. The sharp turning of the southern end of the WSDB occurs in certain months and years. The time of intermittent occurrence should be related to the change in external force, which may be typhoon waves and seasonal currents. The corresponding mechanics for sharp-turn ends are a special topic of island change worth exploring in the future.

### 3.5. Change in the Land Area of the WSDB

Whether WSDB disappears has always been a topic of concern to local residents and the government. The land area surrounded by the 0 m shoreline is a good indicator to illustrate the speed of the disappearance of the WSDB. However, all the waterlines are obtained under different tidal levels, so these are not necessarily the zero-meter shoreline. That is to say, there are almost no cases where the waterline is the zero-meter shoreline. We can select images in which the tidal level at that time only deviates from the mean sea level within ±15 cm. A total of 42 images meet this condition.

**Figure 11:**
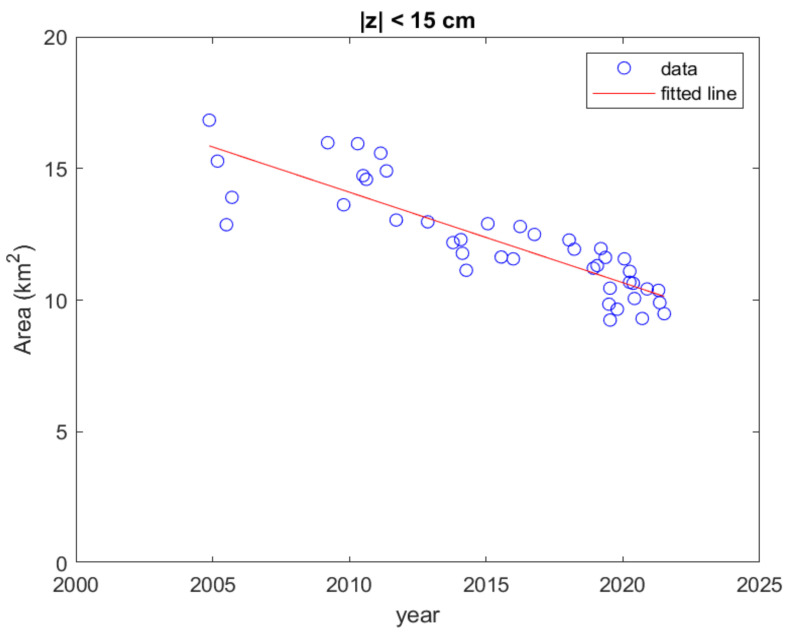
Calculated land area of the entire WSDB for the images chosen for which the tide ranged within ±15 cm.

The attenuation rate of the land area is calculated using linear fitting for these data. The land areas of these images are shown in Figure 11 where the open circles denote the data and the solid line indicates the fitted linear line. To analyze the influence of the number of images selected using the water level range on the estimated attenuation rate of the area, the correlation coefficient (CC) and the root of mean squared error (RMSE) are computed between the obtained area and the estimated area by linear regression. The CC of Figure 11 is 0.864 and the RMSE is 0.99 km^2^. The slope of the descending line is −0.344 km^2^/year to show that the land area of the WSDB is reducing at this attenuation speed. The area reduction rate is close to −0.378 km^2^/year of the previous study [11].

## 4. Discussion

### 4.1. Effect of Tidal Deviation on the Attenuation Rate of the Land Area

**Figure 12:**
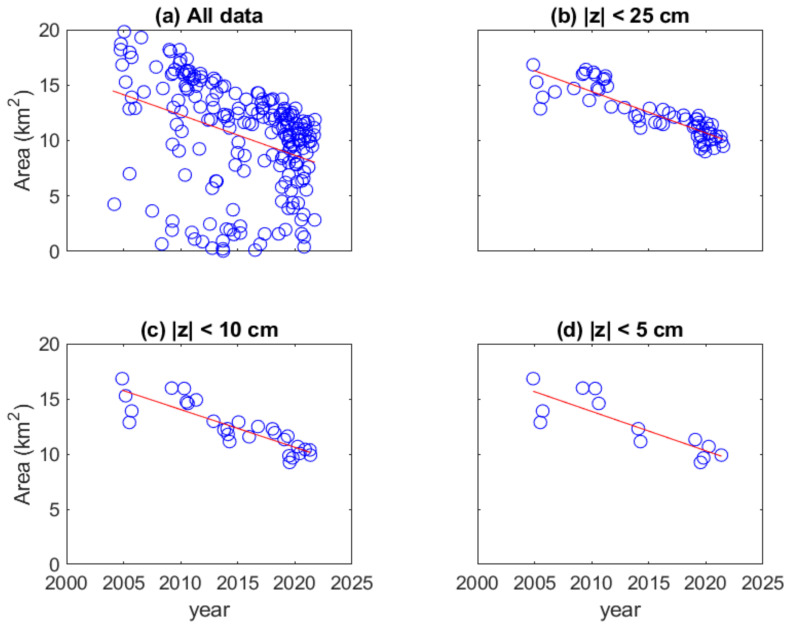
Calculated land area and the corresponding fitting line for four tidal deviation thresholds from the mean sea level. (**a**) maximals; (**b**) 25 cm; (**c**) 15 cm; (**d**) 5 cm. The open circles represent the data and the straight lines are the result of linear regression.

Figure 11 shows the results of the attenuation rate obtained from the land area of 42 images chosen at near mean sea level. If all the land areas of the 207 images are considered, the scattered plot and its linearly fitting line are shown in Figure 12a. Although land areas are widely distributed, the values have a decreasing trend. Its attenuation rate of land area is −0.365 km^2^/year.

As shown in Figure 11, some images are selected in which the tidal levels deviate from the mean sea level. The level deviation is set at 25, 10, and 5 cm, respectively. The number of available images for these three conditions is 61, 29, and 13, respectively. The calculated land area data and the corresponding fitted linear line are shown in Figure 12b–d. The slope of the straight line in Figure 12b–d is −0.375, −0.342, and −0.357 km^2^/year, respectively.

We compare the attenuation rate in Figure 11 with four values in Figure 12. The value in Figure 12c is closest to that in Figure 11. When the range of deviation of the tidal level increases, the land areas become scattered over time, as in Figure 12a,b. The topography of a natural barrier will not be pyramid-shaped and its horizontal cross-sectional area will not have a linear relationship with elevation.

For all images used in Figure 12a, CC is 0.3630 and RMSE is 4.52 km^2^, which is regarded as a low correlation and high RMSE. The result indicates that the estimated decay rate of the land area is biased when the number of images with the tidal level above the mean sea level differs too much from the number of images below the mean sea level. As for the other three panels in Figure 12b–d, the CCs are 0.868, 0.860, and 0.842, respectively. These three values belong to high CC. The CC of Figure 12d is slightly lower than the other two panels. The CC of Figure 12d is slightly lower than the other two panels. The CC of Figure 11 is very close to those of Figure 12 b,c. The RMSEs of Figure 12b–d are 1.06, 1.06, and 1.34 km^2^, respectively. The first two values are almost the same, but slightly higher than the RMSE of Figure 11. Due to a lack of data in some time periods, the number of selected images in Figure 12d is too small to have sufficient data in the regression analysis. The determined decay rate is also unreliable in this case. It is applicable to select images based on the water level deviation of about 15 cm from ZSL to analyze the change trend of the WSDB land area.

### 4.2. Effect of the Tidal Level on the Estimated Land Area

**Figure 13:**
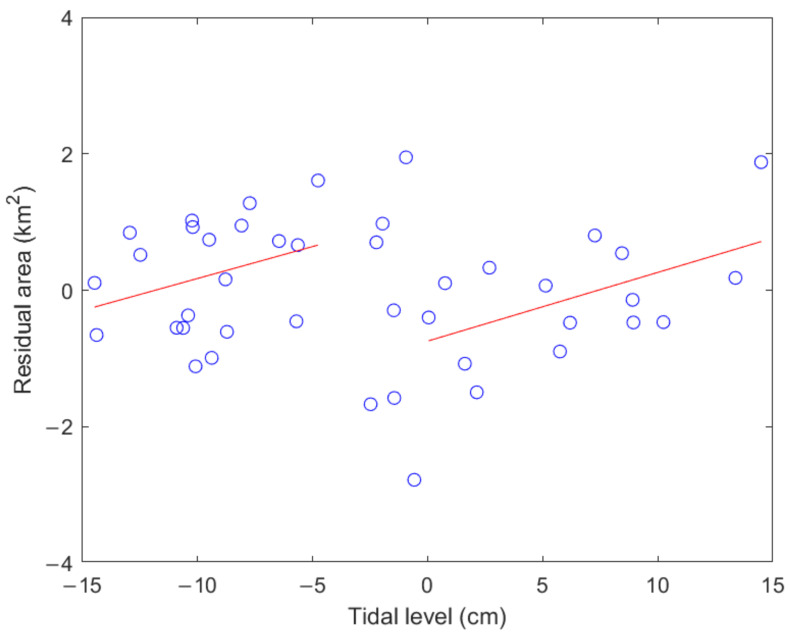
Scatter diagram between residual area and tidal level and tidal level denoted by open circles. Two straight lines are linear regression of partial data at positive or negative water levels.

The attenuation rate in Figure 11 is obtained from 42 images in which the tidal levels range within ±15 cm. The land area surrounded by positive sea level is actually higher than that of negative tidal level. Therefore, it is worth discussing whether the estimated attenuation rate is affected by the tidal level. We subtract the corresponding decaying area from the land area obtained at a certain time, that is, the area value of a circle in Figure 11 minus the value on the straight line at the corresponding time. We call the difference between two kinds of land area a residual area. We plot the estimated residual area against the tidal level in the open circles in Figure 13.

All residual areas in Figure 13 are distributed between ±2 km^2^, and most of them are negative at the positive tidal level, and conversely, most of them are negative at the negative tidal level. Therefore, there is an increasing trend in the positive tidal level parts and the negative tidal level. The correlation coefficient between all residual areas and the corresponding tidal levels is −0.018. However, the correlation coefficients for the positive tidal level and negative tidal level parts are 0.542 and 0.314, respectively. It can be seen that the slight deviation of the tide level from the mean sea level still has a medium or low correlation with the estimate of the land area. When the number of images at positive tide levels is similar to that at negative tide levels, the estimated attenuation rate of the land area is correct regardless of the tide level deviation threshold.

### 4.3. Limitations and Future Developments of SiamUnet

Segmenting terrain types differs from common object segmentation problems. The geological features in satellite images are often monotonous and relatively small in scale. Different geological types have distinct characteristics that can vary with different weather conditions, making it challenging to determine the appropriate size of the sliced patches. Hardware limitations may also restrict the use of larger sliced patches for network construction. To provide the best image resolution to the network while incorporating global information, this study introduced the concept of SiamUnet. This allows for the input of large-scale image information, enabling the SiamUnet to understand the impact of different weather conditions on satellite images without the need for larger sliced patches or extensive exploration of optimal sliced patch sizes for different geological types.

Given the substantial differences in the architectural designs of DeepUnet and SiamUnet, it was deemed inappropriate to conduct an ablation experiment for direct comparison between the two models. Consequently, DeepUnet was utilized as a reference model to demonstrate the enhanced performance capabilities of SiamUnet. This demonstration specifically highlights how SiamUnet, augmented with supplementary information derived from full-frame images, can achieve better results with only 256 × 256 patches.

Although the application of the SiamUnet to coastal areas in this study still required manual adjustments due to the influence of wave foam and land puddles, it effectively accelerated the process of land–sea classification for a large number of images.

The ability to extend the SiamUnet to other satellite images is crucial. In our research, we only used WSDB images as the input dataset of the network. Terrains from different regions may exhibit different image features. To enhance the versatility, it is necessary to collect image data from other regions and explore the generalizability. The large-scale image branch in SiamUnet provides extensive information to the segmentation. In the future, we can further discuss and explore more effective methods to extract information from large-scale images to reduce the overall complexity and size of the network. The code for SiamUnet has been uploaded to GitHub (https://github.com/Sw2chen/SiamUnet (accessed on 2 November 2023)).

## 5. Conclusions

Barrier change is a complex evolutionary process of coastal topography. Accurate analysis of the barrier shows that the zero-meter shoreline provides the change in the shape of the barrier. However, only the waterline difference from the zero-meter shoreline can be extracted from aerial photography, video imagery, or satellite imagery and used for engineering application. The detection of sea–land segmentation separating an image into ocean region and land region is a key technique for accurate detection of water lines in remote sensing imagery. We collected 207 high-resolution satellite images with low cloud coverage from 2004 to 2021.

DeepUNet with high performance in object recognition was considered and trained with different patches. The DeepUNet trained with 256-pixel patches was examined to have poorer model performance than that with a larger size. The SiamUnet was also developed and proven to improve the accuracy of DeepUNet-256 segmentation detection and was shown to be slightly better than DeepUNet-512 and DeepUNet-1024. Barrier waterlines from 207 high-resolution satellite images detected by SiamUnet can show transformation and characteristics of the barrier shape from 2004 and 2021. Cloud cover still affects the segmentation detection by SiamUnet, causing false land segmentation in WSDB. In the future, it is expected that image morphology image post-processing can be used to fill these hollow parts and become land, so as to increase the calculation accuracy of the land area.

Through the detected waterlines of a large number of satellite images, the long-term trend and variation of the movement of the WSDB are clearly visual. We also found two special changes in the shape of the southern end. The first change is the separation from the entire WSDB, which only occurred in 2005, 2006, 2009, 2013–2018, and 2021 when the water level was less than 50 cm. In addition to the time periods mentioned in earlier reports, from 2013 to 2018 using elevation surveying in situ, we identified additional time periods. We also found that the southern separation also occurred at the end of summer in short-term periods, particularly during the summer of 2006. The second change is the southern L-shaped end. Recently, it has shown a zigzag yearly variation since the end of 2017 until 2021. The length gradually decreases in the summer, but gradually increases in the winter. The length of the L-shaped end obviously has a seasonal change.

After computing the land area surrounded by the detected water line for each image, the attenuation rate of the land area is estimated from 42 images at tidal levels within ±15 cm as −0.344 km^2^/year, which slightly varies with the number of images depending on the deviation threshold of the tidal level. However, the slight deviation of the tide level from the mean sea level still has a medium or low correlation with the estimate of the land area. When the number of images at positive tide levels is similar to that at negative tide levels, the estimated attenuation rate of the land area is correct regardless of the tide level deviation threshold.

Single image recognition and object tracking in some previous studies mainly focused on obtaining the described mission. This research mainly builds a network that can identify the boundary between sea and land in satellite images. This Siamese network can be widely used in sea and land identification on sandy beaches. However, if applied to different image sources or landscapes with significant variations, retraining may be necessary.

## Figures and Tables

**Table 1:** The spatial resolution and the number of different satellite images selected.

Satellite	Mode	Spatial Resolution	Number of Selected Images
SPOT-5	Panchromatic	5 m	89
	Supermode	2.5 m	
	Multispectral	10 m	
SPOT-6 and 7	Panchromatic	1.5 m	118
	Multispectral	6 m	

**Table 2:** The IoU statistics of 207 images obtained by four networks.

Statistics	DeepUNet-256	DeepUNet-512	DeepUNet-1024	SiamUnet
std	0.235	0.182	0.187	0.197
Q1	0.432	0.592	0.595	0.584
Q2	0.651	0.717	0.703	0.723
Q3	0.801	0.799	0.799	0.816
max	0.917	0.918	0.924	0.921

## Data Availability

Restrictions apply to the availability of these data. Data was sublicensed by AIRBUS DS from the Center for Space and Remote Sensing Research, National Central University(CSRSR/NCU) and are available at: https://opendata.csrsr.ncu.edu.tw/index.aspx with the permission of CSRSR/NCU.
